# Editorial: Back to the Future: On the Road Towards Precision Psychiatry

**DOI:** 10.3389/fpsyt.2020.00112

**Published:** 2020-02-26

**Authors:** Brisa S. Fernandes, Stefan Borgwardt, André F. Carvalho, Johann Steiner

**Affiliations:** ^1^ IMPACT Strategic Research Centre (Innovation in Mental and Physical Health and Clinical Treatment), School of Medicine, Deakin University, Geelong, VIC, Australia; ^2^ Department of Psychiatry, University of Basel, Basel, Switzerland; ^3^ Department of Psychiatry, University of Lübeck, Lübeck, Germany; ^4^ Department of Psychiatry, University of Toronto and Centre for Addiction & Mental Health (CAMH), Toronto, ON, Canada; ^5^ Department of Psychiatry, University of Magdeburg, Magdeburg, Germany

**Keywords:** precision psychiatry, precision medicine, personalized medicine, biomarker, machine learning, computational psychiatry, mood disorders, schizophrenia

Psychiatry, with the field of Precision Psychiatry, has been experiencing one of the most exciting moments of its history with a paradigmatic shift (1). A few years ago, it became clear that the understanding of psychiatry at the time, mostly descriptive and phenomenologically based, was wanting. This led to a crisis within the psychiatric community, and, some would say, a disbelief in the field; the simply phenomenologically driven paradigm of the time was no longer sufficient.

According to philosophy of science, when a paradigm is threatened by crisis, the community itself, in this case, the psychiatric community, with its clinicians and scientists, is in disarray. For instance, there are moving quotations from Wolfgang Pauli, one a few months before Heisenberg's matrix algebra, which was a major conceptual change in quantum mechanics, and one just some months after. In the former, Pauli expresses the feeling that physics is going to ruin, and he wishes he were in another field; a few months later, the way ahead is clear. At the time, many had the same feeling, and at the height of the crisis, the community was falling apart as the current paradigm was under questioning (2). Crisis and paradigm change go hand in hand; however, crisis does not, in itself, lead to rejection of the existing paradigm. The decision to reject one paradigm is invariable parallel to the decision to accept another, and the logic leading to that decision involves the comparison of both paradigms with each other. In this sense, precision psychiatry, by embracing the heterogeneity of psychiatric disorders, and thus aiming at predicting diagnosis, prognosis, and response to treatment for an unique individual by employing the underlying pathophysiology, as opposed to diagnosing and treating psychiatric disorders merely informed by the somehow subjective clinical characteristics of a patient, constitutes a new paradigm in psychiatry (1).

A new paradigm is always accompanied by exciting new research, and the psychiatric scientific community is becoming fully committed to embracing Precision Psychiatry by actively conducting very high-quality science in the field. By presenting findings from previously separate fields of psychiatry, neurophysiology, and computational modelling, with the Research Topic “*Back to the Future: On the Road Towards Precision Psychiatry*,” we were able to contribute to the advancement and to promote this new field. Precision Medicine has been defined as ‘an emerging approach for treatment and prevention that takes into account each person's variability in genes, environment, and lifestyle' (3). In this Research Topic, research regarding biomarkers and the biosignature of psychiatric disorders was advanced.

Schultze-Lutter et al. calls for an integrative approach and takes the view that only a concise description of psychopathology combined with current neurobiological findings will lead to a breakthrough of more precise diagnostics and therapy.

Some papers of this issue focus on the potential role of immune mechanisms and glial dysfunction in patient subgroups. For instance, Bechter discusses which terminology is best suited for clinical use to adequately categorize cases of mental illness with immune processes affecting the brain. Beyond these theoretical considerations, Kroken et al. identified inflammatory biomarkers that might help in the characterization of distinct biotypes in schizophrenia, and that can be used as potential targets for anti-inflammatory treatments. In addition, Das et al. investigated the potential of Myo-inositol as a biomarker of astroglial dysfunction in schizophrenia that also could be useful for stratification. Still, concerning classification, this time for major depressive disorder, Horne and Foster proposed metabolic and microbiota-based biomarkers, and Walther et al. suggested a hypothesis-free approach with omics, in this case, lipidomics, in tandem with computational psychiatry using machine learning for diagnosis of major depressive disorder. Finally, Gescher et al. suggest that further research in epigenetic modulation may reveal characteristic gene-environmental interactions in different types of personality disorders.

“The right drug for the right patient at the right” time is the core of Precision Psychiatry. This is a very complicated task that that starts with identifying biomarkers for subtyping and classification and reclassification of psychiatric disorders; these newly objectively identified subtypes would lead, in turn, to better pharmacological and psychological interventions. Here enters “the right patient” part of the equation. Suvisaari et al. prosed several biomarkers, including clinical and sociodemographic factors, cognition, brain imaging, genetics, and blood-based biomarkers, to predict different outcomes, such as remission, recovery, and suicide risk in first-episode psychosis, which is necessary for selecting groups with a potentially worse prognosis that would possibly benefit from more aggressive treatment strategies. Joshi and Light proposed an electroencephalography measure called mismatch negativity as a candidate biomarker, and neurocognitive impairment in schizophrenia as a target disease dimension in this context. Amare et al., through polygenic scores, identified eight loci associated with response to Selective Serotonin Reuptake Inhibitors for major depressive disorders. Seeberg et al. suggested that the profile of emotional and non-emotional cognition and neural activity of a given individual, and the early treatment-associated changes in neural and cognitive function, may be useful for guiding treatments for depression. Additionally, Pisoni et al. investigated whether the treatment response of depressive patients could be predicted using growth factor measurements in blood samples. Moving to bipolar disorder, Salagre et al. further refined the concept of staging, as a model capable of enabling Precision Psychiatry in bipolar disorder as a way to categorize patients according to clinical presentation, course, and illness severity, integrating multiple levels of information that can help to define the characteristics, severity, and prognosis of an individual patient in a more individualized manner.

Moving to “the right treatment” part of the equation, Aquino et al. developed a new biosignature, using lipidomics, that could be developed into a blood laboratory test to better guide the antipsychotic choice in schizophrenia, a crucial point in the field, while Davies et al. showed, using meta-analysis, that the currently available interventions for attenuating positive psychotic symptoms in individuals with clinical high-risk for psychosis sadly mostly lack efficacy. Scott et al. argued for the development of a composite consisting of a combination of clinical factors and multimodal biomarkers, such as blood-based omics, neuroimaging, and actigraphy to uncover a valid lithium response phenotype, what would potentially improve eligibility criteria for lithium treatment in bipolar disorder; and Maruani and Geoffroy suggested that bright light therapy can have its efficacy improved by personalizing its regimen according to the pattern of mood disorders, and also if the current depressive episode is a bipolar or unipolar one. Finally, Liu et al. discuss new potential biomarkers of frontotemporal dementia (FTD) subtypes, which could pave the way for individualized approaches, either focusing on tau-targeting (e.g., tau aggregation inhibitors) or progranulin-related therapies. Likewise, for FTD with C9orf72 repeat expansions, candidate antisense therapeutics could be useful.

Other publications in this issue deal with the link of epileptic seizures with psychological and cognitive impairments. In this context, Palanca et al. are aiming to identify which factors may predict cognitive and neurophysiological recovery following electroconvulsive or ketamine treatment in significant depression refractory to pharmacologic therapy. Electroencephalography (EEG) is still useful for differential diagnostic purposes, as illustrated by Endres et al. who present a case with a (para)epileptic manifestation of schizophrenia-like symptoms. However, as summarized by Joshi and Light, more sophisticated EEG measures such as mismatch negativity are also candidate biomarkers for clinical trials.

At the time of publication of our Research Topic “On the Road Towards Precision Psychiatry,” we are in a moment when the academic psychiatry community has joined efforts to move the new field of Precision Psychiatry forward. Slowly, as it always happens when a paradigmatic change is involved, researches in this area are making progress towards better diagnosis and treatment selection for psychiatric disorders. These new advancements will, hopefully, fully unfold in the years to come.

## Author Contributions

All authors contributed to this manuscript.

## Conflict of Interest

The authors declare that the research was conducted in the absence of any commercial or financial relationships that could be construed as a potential conflict of interest.
